# Melatonin Signaling Modulates Clock Genes Expression in the Mouse Retina

**DOI:** 10.1371/journal.pone.0106819

**Published:** 2014-09-09

**Authors:** Susumu Hiragaki, Kenkichi Baba, Elise Coulson, Stefanie Kunst, Rainer Spessert, Gianluca Tosini

**Affiliations:** 1 Neuroscience Institute and Department of Pharmacology and Toxicology, Morehouse School of Medicine, Atlanta, Georgia, United States of America; 2 Institute of Functional and Clinical Anatomy, University Medical Center of the Johannes Gutenberg University, Mainz, Germany; University of Texas Southwestern Medical Center, United States of America

## Abstract

Previous studies have shown that retinal melatonin plays an important role in the regulation of retinal daily and circadian rhythms. Melatonin exerts its influence by binding to G-protein coupled receptors named melatonin receptor type 1 and type 2 and both receptors are present in the mouse retina. Earlier studies have shown that clock genes are rhythmically expressed in the mouse retina and melatonin signaling may be implicated in the modulation of clock gene expression in this tissue. In this study we determined the daily and circadian expression patterns of *Per1*, *Per2*, *Bmal1*, *Dbp*, *Nampt* and *c-fos* in the retina and in the photoreceptor layer (using laser capture microdissection) in C3H-f+/+ and in melatonin receptors of knockout (MT_1_ and MT_2_) of the same genetic background using real-time quantitative RT-PCR. Our data indicated that clock and clock-controlled genes are rhythmically expressed in the retina and in the photoreceptor layer. Removal of melatonin signaling significantly affected the pattern of expression in the retina whereas in the photoreceptor layer only the *Bmal1* circadian pattern of expression was affected by melatonin signaling removal. In conclusion, our data further support the notion that melatonin signaling may be important for the regulation of clock gene expression in the inner or ganglion cells layer, but not in photoreceptors.

## Introduction

Melatonin is synthesized by the pineal gland and the retina of many vertebrate species via a well-defined biosynthetic pathway [1]. Several studies have shown that melatonin synthesis in the retina primarily occurs in the photoreceptors at night [2]–[7]. Experimental evidence indicates that circadian clock controlling melatonin synthesis is located within the photoreceptors. In *Xenopus*, chicken, and rat, rhythmic melatonin synthesis persists in retinae in which the inner retina has been destroyed by kainic acid treatment [3]–[5] or in an isolated photoreceptor layer [6]–[7].

Melatonin exerts its influence by binding to G-protein coupled receptors named melatonin receptor type 1 (MT_1_) and type 2 (MT_2_). MT_1_ and MT_2_ receptors are both present in the vertebrate retina (reviewed in: [8]). In rats MT_1_ receptors are found in the horizontal and amacrine cells, in the inner plexiform layer, retinal ganglion cells, and the retinal pigment epithelium [9]. Dopaminergic neurons may also express MT_1_ receptors [10], suggesting that melatonin can directly modulate the activity of these cells.

In mouse, melatonin receptors have been localized to photoreceptors, inner retinal neurons, and in the ganglion cell layer [11]–[13]. Additional studies have also shown that in mouse, melatonin plays an important role in the modulation of the daily rhythms of visual sensitivity [11]–[13] and affect photoreceptors viability during aging [11]. Finally, it has been recently reported that melatonin acts on the photoreceptors via a MT_1_/MT_2_ receptors heteromer via a Phospholipase C/Protein kinase C (PLC/PKC) pathway [13]. Interestingly the activation of this pathway has been liked to the mechanism by which melatonin can phase-shift circadian rhythms in the SCN [14].

Earlier studies have shown that clock genes are rhythmically expressed in the mouse retina [15]–[16] and a recent investigation has reported that the core circadian clock proteins are expressed in the photoreceptors, but only in the cone photoreceptors do these proteins show a diurnal and circadian variation [17]. Additional investigations have also indicated that melatonin signaling may modulate clock gene expression in the retina [18]. However, it is important to note that this later study compared clock gene expression between a melatonin-proficient (C3H-HeN) and a melatonin-deficient (C57/Bl6) mouse strain [18] and therefore the observed difference in the expression pattern of expression of the clock genes and/or proteins may be also due to other unknown factors. In an additional study [19], the same research team reported that MT_1_^−/−^ and MT_2_^−/−^ knockout mice in a C3H-HeN background showed a significant daily variation in the levels of PER1 and CRY2 in inner and ganglion cell layers. Interestingly the amplitude of the rhythms appeared higher in mice lacking the melatonin receptors than in control mice (i.e., C3H/HeN with melatonin receptors) and removal of melatonin signaling affected the phase of the expression pattern of the PER1 in the inner and ganglion cell layers of MT_1_^−/−^ and in the ganglion cell layer of MT_2_^−/−^ [19].

However, it is important to note that the melatonin-proficient mouse strain used in these studies (C3H-HeN) carries a mutation (*rd/rd*) that leads to a rapid degeneration of the photoreceptors during early post-natal life [20]. Hence this mouse can be useful to study the role of melatonin in the regulation of gene expression in the inner and ganglion cell layers, but not in the photoreceptors.

Our laboratory recently developed melatonin-proficient mice (C3H-f^+/+^) in which the *rd* mutation and the melatonin receptors have been removed [11], [13]. In this study we first investigated the daily and circadian expression pattern of *Period1 (Per1)*, *Period2 (Per2)*, *Bmal1* (*Aryl hydrocarbon receptor nuclear translocator-like*), *Dbp (D site of albumin promoter binding protein)*, *Nampt (Nicotinamide phosphoribosyltransferase)* and *c-fos* in the retina and in the photoreceptors of a C3H-f^+/+^ mice, and then we investigated the effects that melatonin signaling removal produces on the daily and circadian profile of these genes.

## Experimental Procedure

### Animals and sample preparation

Melatonin proficient mice (C3H-f^+/+^; WT [21]) and melatonin proficient mice lacking MT_1_ or MT_2_ receptors were used in this study (C3H-f^+/+^MT_1_^−/−^ [MT_1_^−/−^], and C3H-f^+/+^MT_2_^−/−^ [MT_2_^−/−^]; [13]). The MT_1_^−/−^ and MT_2_^−/−^ mice (C3H-HeN substrain) were backcrossed to C3H-f+/+ (C3H-HeJ substrain) mice for 10 generations to obtain mice of an identical genetic background.

The genotypes were determined according to the protocols previously described [11], [13]. Male and female mice (3–5 months old) were kept in a 12 Light:12 Dark (LD) cycle and were sacrificed starting at Zeitgeber Time (ZT) 1 (i.e., one hour after light onset) and then every 3 hrs over a period of 24 hrs. To measure circadian expression mice were kept in constant darkness (DD) for 60 hrs prior the beginning of the sampling (starting at Circadian Time [CT] 1). During the light phase of the LD cycle, light was supplied by fluorescent tubes (F34CW-RS-WM-ECO, General Electric, Fairfield, CT) with an average intensity ranging from 100–150 µW/cm^2^ at the cage level. The room temperature ranged between 20–23°C and the humidity between 30–70% throughout the whole experiment. Mice were anesthetized by isoflurane and then killed by cervical dislocation.

All the experimental procedures were performed in accordance with NIH Guide on Care and Use of Laboratory Animals and were approved by the Morehouse School of Medicine Animal Care and Use Committee (Protocol number 13–17).

### Retina sampling

After enucleation of the eye, a small incision was performed on the corneal limbus with a sterile blade. The lens and vitreous were discarded, and the retina was directly collected with sterile forceps and immediately frozen on dry ice and stored at −80°C until use. Total retinal RNA was isolated by using TRIZOL Reagent (Life Technologies). RNA was treated with DNAse I (Promega, Fitchburg, WI, USA), and subjected to cDNA synthesis according to the protocol of the manufacturer. Collection of the eyeballs and/or retinas during the dark phase of the LD cycles or DD was done under red dim light (<3 lux, 15 W Kodak safe lamp filter 1A, Eastman Kodak, Rochester, NY, USA). The collection of the retina in LD or DD was performed in less than 1 minute.

### Isolation of photoreceptor layers (PRL)

Whole eyes were embedded in Tissue-Tek OCT (Sakura Finetek USA Inc., Torrance, CA, USA) frozen on dry ice, and stored at −80°C. Frozen tissues were cut into 10-µm-thick sections and mounted on glass slides (VWR Scientific, Radnor, PA, USA) at least six sections obtained from the central part of the retina were used for each eye. The sections were thawed and immediately fixed in 75% ethanol for 30 s, followed by a wash in RNase-free water for 30 s. The sections were then treated with Histogene LCM Frozen Section Staining Kit (Life Technologies Corp., Carlsbad, CA., USA) staining solutions for 45 s, followed by a wash with RNasefree water for 30 s. Finally the sections were dehydrated in graded ethanol solutions (75%, 30 s, 95%, 30 s, and 100%, 30 s) and cleared in xylene (5 min). After being air-dried for 30 min, the slides were kept in a vacuum desiccator for a minimum of 30 min. Laser capture microdissection (LCM) was performed by separately lifting the outer nuclear layer (ONL) onto HS-CapSure non-contact LCM film (Life Technologies Corp.) by using a PixCell IIe LCM system (Life Technologies Corp.). Total RNA was extracted from the captured cells by using the PicoPure RNA Isolation Kit (Life Technologies Corp.). On-column digestion with RNase-Free DNase Set (Qiagen, Venlo, Netherlands) was performed to ensure removal of possible genomic DNA contamination. Samples were reversed transcribed and subjected to RT-PCR analysis as described above (see [2], [7] for further details about the LCM procedure).

### Quantitative Real Time RT-PCR analysis (Q-PCR)

Total RNA was reverse transcribed into first-strand cDNA using a High-Capacity RNA-to-cDNA Kit (Life Technologies Corp.).Q-PCR was performed using the CFX96 Touch Real-Time PCR Detection System (Bio-Rad Laboratories, Hercules, CA, USA) using iQ SYBR Green Supermix (Bio-Rad Laboratories). The efficiency and specificity of the amplification were controlled by generating standard curves and carrying out melting curves, respectively. Primers used were as follows: for *Per1* (GenBank accession number NM_011065), forward, 5′-tgaagcaagaccgggaga-3′ and reverse, 5′-cacacacgccgtcacatcaa-3′ (143 bp product, spanning a ∼480 bp intron); for *Per2* (GenBank accession number NM_011066), forward, 5′-gaaagctgtcaccaccatagaa-3′ and reverse, 5′-aactcgcacttccttttcagg-3′ (186 bp product, spanning a ∼100 bp intron); for *Bmal1* (GenBank accession number NM_007489), forward, 5′-aaccttcccgcagctaacag-3′ and reverse, 5′-agtcctctttgggccacctt-3′ (79 bp product); for *Dbp* (GenBank accession number NM_016974), forward, 5′- cctgaggaacagaaggatga-3′ and reverse, 5′-atctggttctccttgagtcttcttg-3′ (81 bp product); for *Nampt* (GenBank accession number NM_021524), forward, 5′-cataggggcatctgctcatt-3′ and reverse, 5′-gctgctggaacagaatagcc-3′ (120 bp product); for *c-fos* (GenBank accession number NM_010234), forward, 5′-gggacagcctttcctactacc-3′ and reverse, 5′-gatctgcgcaaaagtcctgt-3′ (87 bp product); and for 18S ribosomal RNA (18S rRNA; GenBank accession number MUSRGE51), forward, 5′-ctctgttccgcctagtcctg-3′ and reverse, 5′-ggccgtgcgtacttagacat-3′ (123 bp product). The PCR program was as follows: 10 min at 95°C, followed by 40 cycles of denaturation at 95°C for 15 s and annealing-elongation at 60°C for 1 min. The acquisition of fluorescence data was performed at the end of the elongation step using CFX manager software V 2.1 (Bio-Rad Laboratories). Expression levels of each transcript were normalized by comparison with the amount of *18S* rRNA.

### Data Analysis

Results are presented as mean ± standard error of the mean (SEM). Cosinor analysis [22] was done using the nonlinear regression model within Sigmaplot V 10.0 (Systat Software, San Jose, CA, USA) was used to assess rhythmicity of gene expression and to fit a cosine curve to the gene expression data. The model can be written according to the equation: f(x) = A+B cos [2 π(x) C) / 24] with the f(x) indicating relative expression levels of target genes, x indicating the time of sampling (h), A indicating the mean value of the cosine curve (mesor; midline estimating statistic of rhythm), B indicating the amplitude of the curve (half of the sinusoid) and C indicating the acrophase (h). Transcript levels were calculated relative to the average expression of each dataset throughout 24 hrs to plot temporal expression. The level of significance for all tests was set at *p*<0.05. In addition to the cosinor analysis we also analyzed the data using CircWave, which can be found at (http://www.rug.nl/fwn/onderzoek/programmas/biologie/chronobiologie/downloads/index).

## Results

### Daily and circadian rhythms in clock and clock-controlled gene expression in mouse retina of WT, MT_1_^−/−^ and MT_2_^−/−^

**Figure 1:**
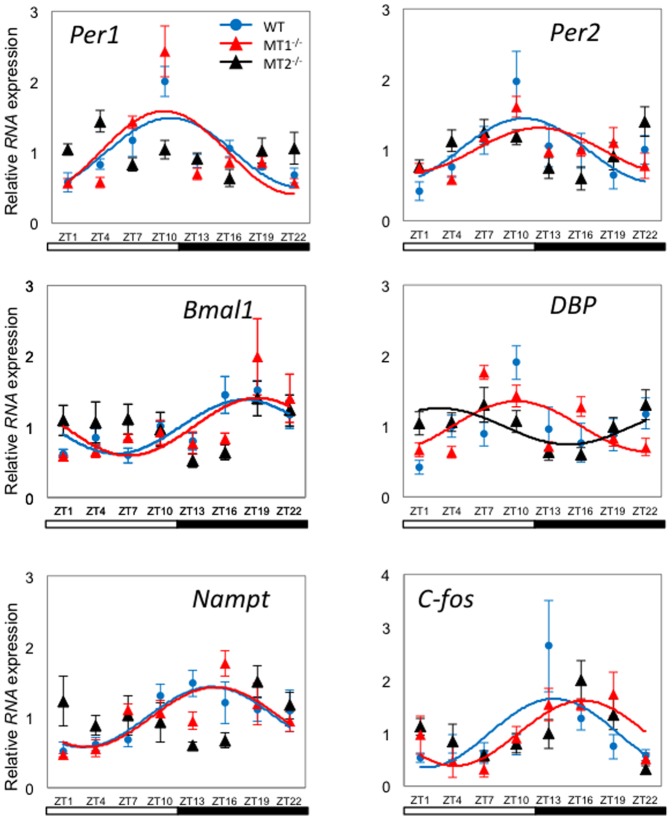
Expression profile of clock genes (*Per1*, *Per2* and *Bmal1*) and clock-controlled gene (*Dbp*, *Nampt*, *c-fos*) transcripts in mouse retina under a LD condition. Blue circle (WT), red triangle (MT_1_^−/−^), and black triangle (MT_2_^−/−^) indicate expression levels of target genes calculated relative to the average expression of each dataset throughout 24 hrs, and which was rescaled to one. Results are expressed as mean ± SEM. Blue (WT), red (MT_1_^−/−^), and black (MT_2_^−/−^) lines represent the periodic sinusoidal functions determined by cosinor analysis (*p*<0.05, (N = 4–6 for each time-point and genotype).

Daily profiles of *Per1*, *Per2*, *Bmal1*, *Nampt, Dbp*, and *c-fos* mRNA were analyzed in mouse retinas of WT, MT_1_^−/−^ and MT_2_^−/−^ (Table 1 and Figure 1). Cosinor and CirWave analysis confirmed statistically significant daily rhythmicity of *Per1*, *Per2*, *Bmal1*, *Nampt*, and *c-fos* mRNA in the retina of WT and MT_1_^−/−^ mice. In all cases the amplitude of the rhythm was small (Table 1). In most case, clock genes and clock-controlled genes peaked at the same time in retina of WT and MT_1_^−/−^ mice. *Dbp* showed significant rhythmicity in the retina of MT_1_^−/−^ and MT_2_^−/−^ mice, but not in WT. *Per1* and *Per2* transcripts peaked at about ZT10, *Bmal1* peaked at midnight, and *Nampt* peaked about ZT15 in WT and MT_1_^−/−^ mice. In MT_2_^−/−^ only *Dbp* mRNA showed a significant daily rhythm (Table 1 and Figure 1).

**Table 1:** Mesor, amplitude and Acrophase (± SEM) of mRNA levels of clock and clock-controlled genes in the retina of WT, MT_1_^−/−^, MT_2_^−/−^ mice under LD or DD conditions.

		mesor			amplitude			acrophase (h)			*P*-value	mesor			amplitude			acrophase (h)			*P*-value
		*Per 1*										*Per 2*									
LD	WT	0.98	±	0.07	0.48	±	0.09	10.51	±	0.78	<0.01	1.00	±	0.09	0.45	±	0.13	10.70	±	1.13	<0.01
	MT_1_^−/−^	0.65	±	0.05	0.38	±	0.08	9.95	±	0.75	<0.01	1.22	±	0.06	0.39	±	0.09	12.12	±	0.89	<0.01
	MT_2_^−/−^		—			—			—		n.s.		—			—			—		n.s.
DD	WT	1.01	±	0.06	0.63	±	0.08	12.44	±	0.47	<0.01	1.01	±	0.07	0.76	±	0.10	12.60	±	0.49	<0.01
	MT_1_^−/−^	2.56	±	0.17	1.82	±	0.24	6.50	±	0.51	<0.01	1.80	±	0.13	1.44	±	0.19	6.93	±	0.50	<0.01
	MT_2_^−/−^	0.71	±	0.05	0.23	±	0.07	6.03	±	0.18	<0.01	0.26	±	0.01	0.12	±	0.02	7.73	±	0.68	<0.01
		*Bmal 1*										*Dbp*									
LD	WT	1.03	±	0.06	0.39	±	0.08	17.88	±	0.76	<0.01		—			—			—		n.s.
	MT_1_^−/−^	0.58	±	0.06	0.24	±	0.08	19.09	±	1.30	<0.05	0.58	±	0.03	0.21	±	0.05	10.05	±	0.91	<0.01
	MT_2_^−/−^		—			—			—		n.s.	0.35	±	0.02	0.09	±	0.03	2.85	±	1.28	<0.05
DD	WT	1.00	±	0.06	0.49	±	0.08	13.33	±	0.64	<0.01	1.02	±	0.06	0.74	±	0.08	11.12	±	0.42	<0.01
	MT_1_^−/−^	2.44	±	0.22	1.57	±	0.31	5.85	±	0.74	<0.01	2.46	±	0.14	1.47	±	0.20	5.50	±	0.52	<0.01
	MT_2_^−/−^	0.45	±	0.03	0.11	±	0.04	3.30	±	1.50	<0.05	0.60	±	0.04	0.34	±	0.06	7.39	±	0.71	<0.01
		*Nampt*										*c-fos*									
LD	WT	1.03	±	0.07	0.44	±	0.10	14.78	±	0.82	<0.01	1.00	±	0.14	0.65	±	0.19	13.47	±	1.13	<0.01
	MT_1_^−/−^	0.89	±	0.06	0.38	±	0.08	15.17	±	0.84	<0.01	0.84	±	0.08	0.52	±	0.12	16.19	±	0.85	<0.01
	MT_2_^−/−^		—			—			—		n.s.		—			—			—		n.s.
DD	WT	1.02	±	0.05	0.63	±	0.07	12.55	±	0.44	<0.01	1.06		0.17	1.05		0.25	13.16	±	0.84	<0.01
	MT_1_^−/−^	3.47	±	0.25	1.55	±	0.35	5.09	±	0.88	<0.01	0.96		0.09	0.35		0.12	10.58	±	1.37	<0.05
	MT_2_^−/−^	0.36	±	0.02	0.14	±	0.03	6.82	±	0.85	<0.01		—			—			—		n.s.

*In this set of data CircWave detected a significant rhythm.

**Figure 2:**
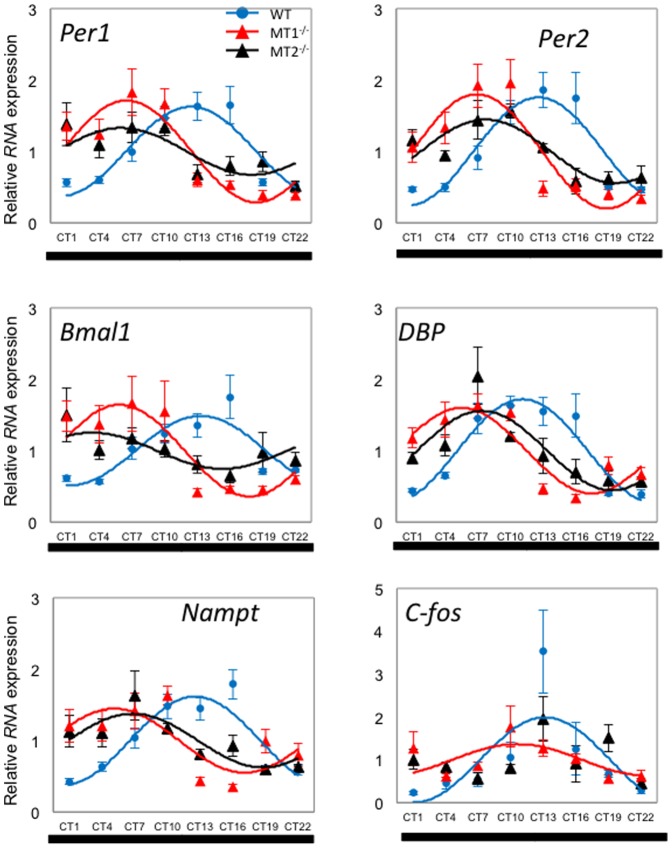
Expression profile of clock genes (*Per1*, *Per2* and *Bmal1*) and clock-controlled gene (*Dbp*, *Nampt*, *c-fos*) transcripts in mouse retina under DD condition. Blue circle (WT), red triangle (MT_1_^−/−^), and black triangle (MT_2_^−/−^) indicate expression levels of target genes calculated relative to the average expression of each dataset throughout 24 hrs, and which was rescaled to one. Results are expressed as mean ± SEM. Blue (WT), red (MT_1_^−/−^), and black (MT_2_^−/−^) lines represent the periodic sinusoidal functions determined by cosinor analysis (*p*<0.05, (N = 4-6 for each time-point and genotype).

In DD conditions, most clock and clock-controlled gene transcript showed circadian rhythmicity in all the three genotypes (Table 1 and Figure 2). In WT *Per1* and *Per2* peaked at around CT 12.5, *Bmal1* peaked at around CT 13, *Dbp* peaked at around CT 11, and *Nampt* and *c-fos* peaked at around CT 13 in WT mice. *c-fos* mRNA showed a significant rhythmicity in WT and MT_1_^−/−^, but not in MT_2_^−/−^ (Table 1 and Figure 2). Interestingly the phase of *Per1, Per2, Bmal1, Dbp and Nampt* was significantly affected (about 6 hrs) by removal of melatonin signaling (Table 1) and under DD the amplitude of the mRNA rhythm for most of the genes was larger than what observed in LD (Table 1).

### Daily and circadian rhythms in clock and clock-controlled gene expression in the photoreceptor layers of WT, MT_1_^−/−^ and MT_2_^−/−^

**Figure 3:**
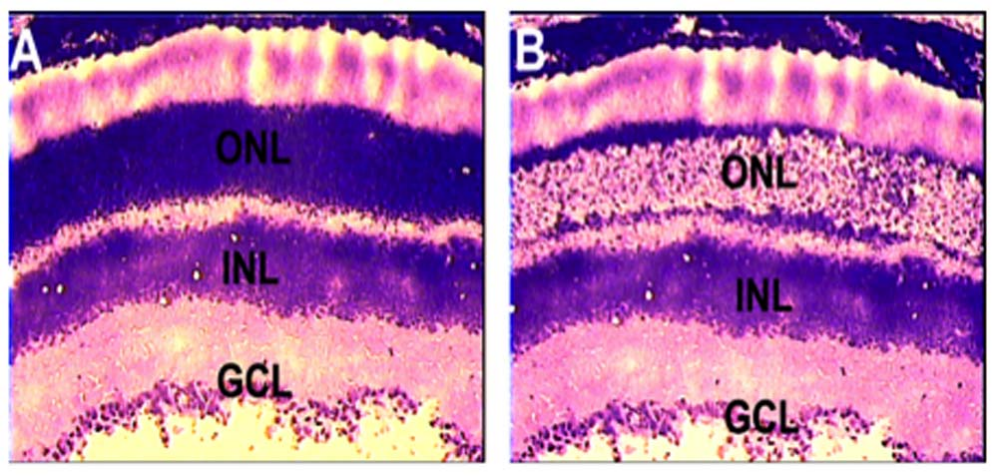
Photomicrograph of retinal section of before (A) and after (B) laser microdissection of the outer nuclear layer (ONL). Ganglion cell layer (GCL); Inner nuclear layer (INL). See Materials and Methods section for more details about LCM.

**Figure 4:**
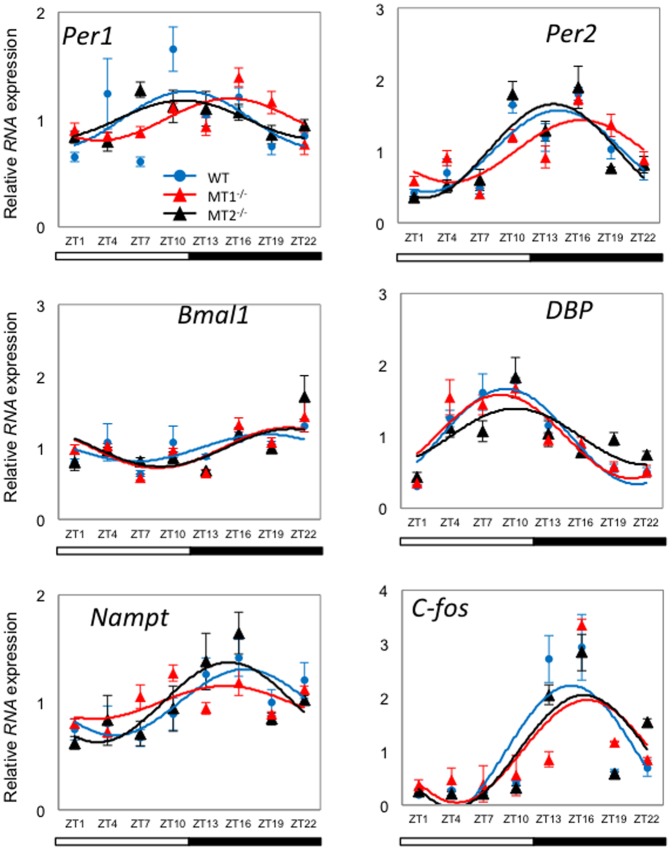
Expression profile of clock genes (*Per1*, *Per2* and *Bmal1*) and clock-controlled gene (*Dpb*, *Nampt*, *c-fos*) transcripts in mouse photoreceptor cell layer under a LD condition. Blue circle (WT), red triangle (MT_1_^−/−^), and black triangle (MT_2_^−/−^) indicate expression levels of target genes calculated relative to the average expression of each dataset throughout 24 hrs, and which was rescaled to one. Results are expressed as mean ± SEM. Blue (WT), red (MT_1_^−/−^), and black (MT_2_^−/−^) lines represent the periodic sinusoidal functions determined by cosinor analysis (*p*<0.05, N = 3–4 for each time-point and genotype).

**Figure 5:**
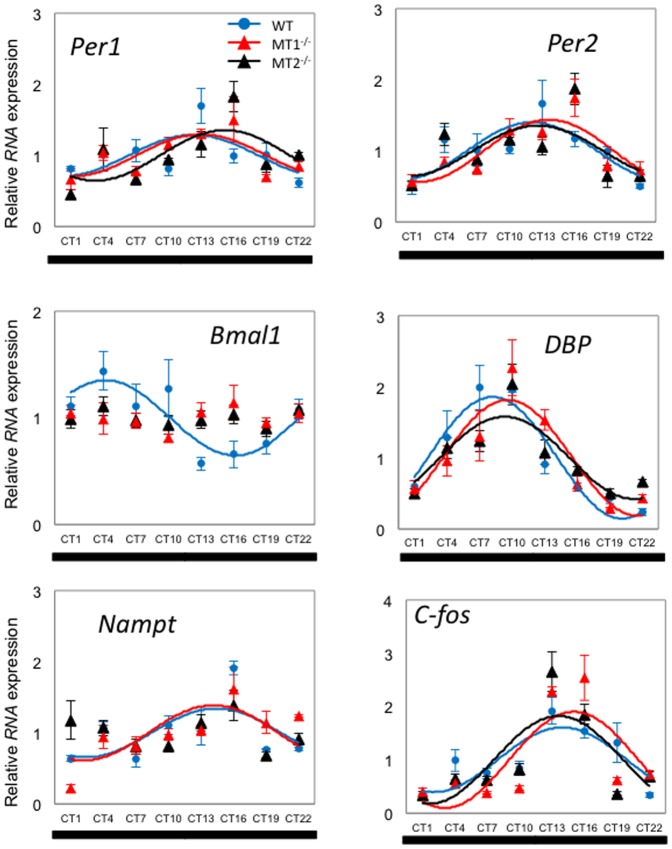
Expression profile of clock genes (*Per1*, *Per2* and *Bmal1*) and clock-controlled gene (*Dbp*, *Nampt*, *c-fos*) transcripts in mouse photoreceptor cell layer under a DD condition. Blue circle (WT), red triangle (MT_1_^−/−^), and black triangle (MT_2_^−/−^) indicate expression levels of target genes calculated relative to the average expression of each dataset throughout 24 hrs, and which was rescaled to one. Results are expressed as mean ± SEM. Blue (WT), red (MT_1_^−/−^), and black (MT_2_^−/−^) lines represent the periodic sinusoidal functions determined by cosinor analysis (*p*<0.05, N = 3–4 for each time-point and genotype).

To investigate the pattern of expression of *Per1, Per2, Bmal1, Dbp, Nampt* and *c-fos* mRNA we performed Q-PCR with cells obtained from the PRL using LCM (see Figure 3). The daily pattern of expression of *Per1*, *Per2*, *Bmal1*, *Nampt, Dbp, Nampt* and *c-fos* mRNAs showed low amplitude, but significant, rhythms in the three genotypes (Table 2, Figure 4). In DD *Per1*, *Per2*, *Dbp, Nampt* and *c-fos* showed low amplitude circadian rhythms and, differently from what observed in the retina, removal of melatonin signaling did not affect the phase of the rhythms of these genes (Table 2, Figure 5). Surprisingly *Bmal1* mRNA was not rhythmically transcribed in the PRL of MT_1_^−/−^ and MT_2_^−/−^ mice (Table 2, Figure 5).

**Table 2:** Mesor, amplitude and Acrophase (±SEM) of mRNA levels of clock and clock-controlled genes in the photoreceptors dissected by laser capture microdisection of WT, MT_1_^−/−^, MT_2_^−/−^ mice under LD or DD conditions (see data analysis section for details).

		mesor			amplitude			acrophase (h)			*P*-value	mesor			amplitude			acrophase (h)			*P*-value
		*Per 1*										*Per 2*									
LD	WT	1.00	±	0.07	0.26	±	0.10	11.25	±	1.45	<0.05	1.00	±	0.07	0.57	±	0.09	14.18	±	0.62	<0.01
	MT_1_^−/−^	0.84	±	0.03	0.16	±	0.04	15.22	±	0.96	<0.01	0.96	±	0.06	0.41	±	0.08	16.26	±	0.73	<0.01
	MT_2_^−/−^	0.61	±	0.02	0.11	±	0.03	10.89	±	1.16	<0.01	1.13	±	0.08	0.74	±	0.11	13.76	±	0.61	<0.01
DD	WT	1.00	±	0.06	0.29	±	0.09	12.31	±	1.15	<0.01	1.00	±	0.07	0.40	±	0.09	12.08	±	6.62	<0.01
	MT_1_^−/−^	1.40	±	0.08	0.42	±	0.11	13.04	±	1.04	<0.01	1.45	±	0.08	0.63	±	0.12	13.63	±	0.72	<0.01
	MT_2_^−/−^	1.18	±	0.09	0.42	±	0.12	15.22	±	1.11	<0.05	1.09	±	0.08	0.39	±	0.12	12.62	±	1.15	<0.05
		*Bmal 1*										*Dbp*									
LD	WT	1.00	±	0.04	0.19	±	0.06	18.55	±	1.27	<0.05	1.02	±	0.05	0.68	±	0.07	9.14	±	0.39	<0.01
	MT_1_^−/−^	1.17	±	0.05	0.33	±	0.08	20.58	±	0.87	<0.01	1.10	±	0.06	0.65	±	0.09	8.64	±	0.53	<0.01
	MT_2_^−/−^	0.87	±	0.05	0.23	±	0.07	20.85	±	1.26	<0.05	1.28	±	0.08	0.50	±	0.12	9.99	±	0.91	<0.05
DD	WT	1.00	±	0.06	0.35	±	0.08	4.16	±	0.87	<0.01	1.00	±	0.07	0.86	±	0.10	8.19	±	0.44	<0.01
	MT_1_^−/−^		—			—			—		n.s.	0.81	±	0.07	0.66	±	0.10	9.59	±	0.55	<0.01
	MT_2_^−/−^		—			—			—		n.s.	0.54	±	0.03	0.32	±	0.05	9.30	±	0.59	<0.01
		*Nampt*										*c-fos*									
LD	WT	1.00	±	0.05	0.31	±	0.07	16.58	±	0.90	<0.05	1.00	±	0.15	1.23	±	0.21	15.03	±	0.65	<0.01
	MT_1_^−/−^	1.16	±	0.04	0.18	±	0.05	14.77	±	1.17	<0.05	0.83	±	0.11	0.79	±	0.16	16.49	±	0.75	<0.01
	MT_2_^−/−^	1.03	±	0.07	0.38	±	0.09	15.07	±	0.94	<0.01	1.29	±	0.16	1.35	±	0.22	16.18	±	0.62	<0.01
DD	WT	1.00	±	0.07	0.34	±	0.10	14.41	±	1.09	<0.01	1.00	±	0.08	0.61	±	0.12	13.95	±	0.74	<0.01
	MT_1_^−/−^	0.58	±	0.04	0.23	±	0.06	14.14	±	1.00	<0.01	0.90	±	0.10	0.81	±	0.14	15.02	±	0.66	<0.01
	MT_2_^−/−^		—			—			—		n.s.	0.97	±	0.10	0.80	±	0.15	13.60	±	0.70	<0.01

Interestingly we only found one set of data out of seventy-two in which the cosinor analysis and CircWave gave different results. In MT_2_^−/−^ knockout under DD conditions retinal *c-fos* levels were not rhythmic according to the cosinor analysis, whereas they were rhythmic using CircWave.

## Discussion

The aim of this study was to investigate the daily and circadian pattern of expression of three clock genes (*Per1, Per2 and Bmal1*) and three clock-controlled genes (*c-fos, Nampt, Dbp*) in the retina and in PRL of a melatonin-proficient mouse (C3H/f^+/+^) strain and then in mice lacking melatonin receptors. Our data indicated that most of these genes were rhythmical regulated in LD and DD in the retina and in the PRL. In our study we focused on *Per1, Per2 and Bmal1* since these clock genes are believe to be a core component of the circadian clock and previous studies have reported that these genes are rhythmically transcribed in the retina [15]–[16]. Similarly, we focused on *Dbp*, *c-fos* and *Nampt* since these genes are clock control genes and may play an important role in the modulating of photoreceptor viability and metabolism [23]–[25].

The results obtained in the WT retina (Figure 1, 2 and Table 1) well agree with those obtained in two previous studies of C57/BL6 mice [15]–[16]. This result is not a complete surprise since it has been reported that C57/BL6 may also produce a small amount of melatonin for a brief period during the night [26]–[27] and therefore is possible that in these so-called melatonin deficient mice the MT_1_ and MT_2_ receptors may also be activated by this small amount of melatonin. A previous study using melatonin receptor knockout mice in a C3H-HeN genetic background (i.e., a strain in which photoreceptors degenerate early in the post-natal life) has also reported that melatonin signaling affects the rhythmicity of clock genes and proteins in the inner retina via the MT_1_ receptors, whereas in the ganglion cell layers both the MT_1_ and MT_2_ receptors seen to affect the expression of these protein [19]. Our data only partially agree with this previous study since in our investigation significant changes in gene expression were mostly observed in MT_2_ knockout mice (Figure 1 and Table 1).

Although these studies have provided important data on the expression of clock genes in the retina, it is worthwhile to mention that this approach is not likely to provide much insight into the functioning of the retinal circadian system since previous studies have shown that clock genes may be expressed in different cell types, and possibly, with different phases (see [28] for a recent review).

To gain a better understanding of clock gene expression in the retina, recent investigations have used LCM combined with Q-PCR to study gene expression in a specific retinal layer. In the first study, it was reported that most of the clock genes are present in the rat PRL [7], then a second study reported that the mRNA levels for *Clock, Bmal1, Per1, Per3, Cry2* and *Casein kinase Ie* had variation over 24-hours in rats maintained in LD cycle, whereas in DD only *Clock* and *Per3* showed a significant rhythm [29]. Finally, a third study using the same experimental approach mostly confirmed the previous work [30], thus suggesting that in the rat the core clock genes are indeed present in the PRL where they are rhythmically expressed when the rat are maintained in LD cycles, but these genes are no longer rhythmically transcribed when the rat are maintained in DD.

A similar approach has been also recently used in the mouse retina and the results obtained are different from those reported for the rat. In PRL obtained from mice held in DD, most of the clock genes are rhythmically expressed in the photoreceptors [31]; unexpectedly the circadian rhythm in gene expression was lost in mice lacking melanopsin (*Opn4* knock-out) [31]. This suggests that melanopsin and its signaling is somewhat involved in the functioning of the clockwork in the photoreceptors.

Our data obtained with the PRL obtained from mice held in DD partially agrees with those reported by Dkhissi-Benyahya et al., [29] since we also detected a circadian rhythm in the expression in *Per1*, *Per2*, *Bmal1*, *Dbp*, but only *Bmal1* and *Dbp* showed a similar phase (acrophase, Table 2). A possible explanation for this disagreement can be found in the observation that the studies by Dkhissi-Benyahya et al., [29] used a C57/BL6, while our investigation was done in C3H-f^+/+^ mice (see previous paragraph about melatonin production in C57/BL6 and C3H-f^+/+^ mice).

Previous studies have demonstrated that rhythmic expression of the clock gene *Per1* in the pituitary gland depends on the heterologous sensitization of the adenosine A_2b_ receptors via the activation of MT_1_ signaling during the night [32] and additional studies have reported that the rhythmic expression of several other clock genes (*Per1, Per 2, Bmal1, and Cry 1*) in the mouse *pars tuberalis* depend on MT_1_ signaling as well [33]. Melatonin signaling, probably via MT_2_ receptors, has been also implicated in the regulation of PER1 and CRY1 in the SCN [34].

As previously mentioned, a similar role for melatonin signaling has been also proposed for the retina since it has been reported that melatonin may influence circadian clock gene expression in the retina since the amplitude and the phase of *Per1, and Cry1 m*RNA and protein in the mouse retina is different between melatonin-proficient, melatonin-deficient mice, and mice lacking melatonin signaling [18]–[19].

Our data confirm these studies in demonstrating that removal of melatonin signaling produces significant effects on gene expression in the retina (Figure 1 and 2, Table 1), but not in the PRL (Figure 4 and 5, Table 2). Interestingly, it appears that in LD, the removal of MT_2_ signaling produces an arrhythmic pattern of expression in *Per1*, *Per2 Bmal1 Nampt* and *c-fos*, thus suggesting that MT_2_ signaling is important for the regulation of clock gene expression in the inner retina. Our data also indicate that the removal of melatonin induces a significant change in the phase (about six hours) in *Per1, Per2, Bmal1, Dbp and Nampt*, but not in *c-fos* (Table 2), thus indicating that under DD conditions melatonin signaling is important for regulating the expression of clock genes in the inner and ganglion cell layers. Further studies will be required to identify the genes expressed in various specific cell types within the inner and ganglion cell layers, and how melatonin signaling may affect the pattern of expression of these genes. In our study we did not use mice lacking both melatonin receptors (i.e., MT_1_^−/−^MT_2_^−/−^) and therefore we cannot exclude that the lack of both receptors may have produced a stronger phenotype. However it must be mentioned that - since the action of melatonin on the photoreceptor cells is mediated by a melatonin receptors heteromer [13] – it is very likely that PRLs obtained from double knock-out mice would have produced a similar result of those obtained with PRLs obtained from MT_1_^−/−^ and MT_2_^−/−^ mice. Finally unpublished data obtained in our laboratory with MT_1_^−/−^MT_2_^−/−^ indicate that these mice have a similar phenotype of that reported for MT_1_^−/−^ or MT_2_^−/−^ at least with respect to visual processing and retinal cells viability.

The results obtained with the PRL were somewhat unexpected since melatonin receptors are expressed in the mouse photoreceptors [11]–[13] and therefore we expected a strong effect on the regulation of clock and clock-controlled genes in melatonin receptor knockout mice. As shown in Figure 4, 5 and Table 2, only *Bmal1* mRNA expression was affected by melatonin signaling removal since it was no longer rhythmic under DD conditions. We believe that this unexpected result can be explained by two alternative hypotheses. First, it is possible that the functioning of the photoreceptor circadian clock does not require rhythmic expression of *Bmal1* or alternatively the expression of clock genes in the PRL is driven by a neurohumoral signal (e.g., dopamine or GABA) from the inner retina [35].

The outer nuclear layer contains the nuclei of the rod and the cone photoreceptors with the rods being the vast majority of the cell (about 95 to 97%) and therefore it could be assumed that the gene expression patterns that we have observed mostly represent the transcription pattern of these genes in rod photoreceptors. However, as we have previously mentioned, it appears that rod photoreceptors may not express clock genes [15], whereas it has been reported that the core circadian clock proteins (CLOCK, BMAL1, NPAS2, PERIOD1, PERIOD2 and CRYPTOCHROME 2) are expressed only in the cone photoreceptors where these proteins show a diurnal and circadian variation [17]. Therefore, it is likely that our data also describe the expression of these clock genes in the cone photoreceptors.

In conclusion, our data further support the notion that clock genes are rhythmically expressed in the photoreceptors in LD and DD, and contrary to our expectation, their pattern of expression is minimally affected by removal of melatonin signaling. On the other end, our data also indicate that melatonin signaling may be important for the regulation of clock gene expression in the inner or ganglion cell layer. Further studies using a similar approach of that used by Liu et al., [17] will be required to identify which cell within the inner and ganglion cell layers express clock genes and proteins and in which cells the expression pattern of the clock genes is affected by melatonin.
